# Pennsylvania State Medical Association

**Published:** 1894-09

**Authors:** 


### PENNSYLVANIA STATE VETERINARY MEDICAL ASSOCIATION.

The Pennsylvania State Veterinary Medical Association assembled in the College of Physicians, Thirteenth and Locust Streets, President Hoskins in the chair. The meeting was called to order at 10:30 A.M. At roll call the following members were present: Drs. Allen, Benner, Bridge, Collins, Conard, Custer, Ferley, Formad, Gladfelter, Glass, Goentner, Harger, Walter L. Hart, John R. Hart, Hoskins, Houldsworth, Keely, Keil, Kooker, Lusson, McCarthy, McNeil, Pearson, Thomas B. Rayner, James B. Rayner, Ridge, Sallade, Schaufler, Schrieber, Senseman, Webster, Williams and Zuill.

There were also present: Prof. Adams, of the Veterinary Department University of Pennsylvania; Drs. McAnulty; E. S. Moyer; B. Lee, State Board of Health; Ritter and May. As delegates from New Jersey State Veterinary Medical Association, Drs. Dustan and Lockwood, and a number of students from the University of Pennsylvania.

Letters of regret were received from Drs. Lee and Waugh, Mr. Beitler, Director of Public Safety of Philadelphia, and Secretary Edge, of the Board of Agriculture.

The minutes of the September meeting were then read by the Secretary, and on motion adopted as read.

The President then read his address, which was a brief review of the progress made in Association circles, and the work accomplished by this Association the past two years; referring to the trying times in all avocations he gave words of encouragement to all those who would remain steadfast to the true aims and objects of the profession during the present trying crisis.

The Chair announces that nominations for officers is now in order, when the following nominations were made: President, Leonard Pearson; First Vice-President, W. H. Ridge; Second Vice President, Thos. B. Rayner; Third Vice-President, Drs. Keil and Waugh; Recording Secretary, W. G. Benner; Corresponding Secretary, Drs Helmer, Ridge, Harger, Allen, Gladfelter, Goentner; Treasurer, John R. Hart; Board of Censors. Drs. Harger, Zuill. Hoskins, Bridge, Kooker, Thos. B. Rayner, McNeil.

There was a motion put that the rules be suspended and the Secretary cast the ballot for nominees where there was no opposing candidate. There being some objection the Chair ruled that a full ballot be cast. Drs Jas. B. Rayner and Webster being appointed by the Chair as tellers, the election was proceeded with, and the tellers then reported the tally of ballot.

President, Leonard Pearson, 22 votes; First Vice-President W. H. Ridge, 22

votes; Second Vice-President, Thos. B. Rayner, 22 votes; Third Vice-President, Jas. A. Waugh 16 votes, and Z. S. Keil 5 votes; Recording Secretary, W. G. Benner, 21 votes; Corresponding Secretary, W. H. Ridge 2 votes, F. S. Allen 12 votes, R. Gladfelter 2 votes, C. Goentner 7 votes; Treasurer, John R. Hart 20 votes; Board of Censors, S. J. J. Harger 21 votes, W. L. Zuill 15 votes, F. Bridge, 10 votes; W. S. Kooker 16 votes, J. C. McNeil 19 votes, W. H. Hoskins 18 votes, Thos. B. Rayner 14 votes, Jas. B. Rayner 2 votes.

The tellers were requested to recount the votes, as they had counted two ballots cast for Jas. B. Rayner to account of Thos. B. Rayner, the above being the corrected count of tellers.

The President then thereupon announces the elected officers for the ensuing year: President, Leonard Pearson; First Vice-President, W. H. Ridge; Second Vice-President, Thos. B. Rayner; Third Vice-President, Jas. A. Waugh; Recording Secretary, W. G. Benner; Corresponding Secretary, F. S. Allen; Treasurer, John R. Hart; Board of Censors, S. J. J. Harger, Chairman ; J. C. McNeil, W. Horace Hoskins, W. S. Kooker and W. L. Zuill.

Corresponding Secretary Ridge then read the following applications for membership: Dr. W. A. Shields, V.M.D., Dr. Charles Ernest, V.M.D., Prof. John W. Adams, V M.D., Dr. E. S. Moyer and Dr. James McAnulty, also those which had been laid over of Drs. Tomlinson, Cawley and Fox.

A recess of fifteen minutes was then taken that the Board of Trustees might examine into the aforesaid applications.

The Board being prepared to report the gavel from the Chair restored order, and the report of Censors was given by Prof. Harger offering a favorable recommendation of Drs. Shields, Adams, Ernest, Moyer and McAnulty. Unfavorably recommended Drs. Tomlinson, Fox and Cawley.

The report of Board of Trustees was favorably received.

The Chair then directs that each applicant favorably reported by the Board shall be balloted for. A motion prevailed, however, that applicants shall be elected singly by acclamation. The election of applicants was so conducted, and Drs. W. A. Shields, Chas. M. Ernest, John W. Adams, Jas. McAnulty and E. S. Moyer were elected to membership. The President then introduced those of the elected members which were present.

A 1 o'clock a motion was then entertained to adjourn for luncheon at "Boothby's Restaurant," the same being extended by the Philadelphia members to all present, who heartily enjoyed the same.

The afternoon session was called to order at 2.15 P. M.

The Chair announces that the delegates of the New Jersey Veterinary Medical Association, Drs. Dunstan and Lockwood, were present, and a cordial welcome with privileges of the floor was extended them. The Chair further states that 2 P. M. was the time appointed to open the discussion and consider the advisability of continuing the attempted legislation relative to milk and meat inspection, and that the letters of regret from Secretary Edge and Director Beitler were a disappointment to himself and all the members, as much information upon this subject was expected from them. He requests that Prof. Pearson open the subject, which he did, and after stating in what form the bill had been presented to the Legislature, thought that it might in some way be improved upon, when we could hope for better success in the future. He laid considerable stress upon the importance of eradicating tuberculosis, and made many valuable suggestions which were edifying to all present.

Dr. Benj. Lee, from the State Board of Health, was the next speaker upon the subject, and gave also many valuable suggestions. He thinks the standard of solids might be somewhat reduced, which would strengthen the bill of milk inspection, as the reduction from 12 per cent. to 11 per cent. would make little difference to the health of the general public, and would give the bill a better chance to pass in this respect. He referred to an act which was passed by the Massachusetts Legislature, when in May and June the standard of milk was lower there those months owing to the fact that animals grazed upon early vegetation when it contained much juice, and in consequence would not yield such rich milk as when they are more matured. He spoke earnestly of the importance of eradicating tuberculosis in domesticated animals, the products of which were used for human food, and says that the State of Massachusetts has gone at it in earnest; that ten thousand dollars ($10,000) were appropriated in that State for laboratory work alone, and their entire appropriation amounted to forty thousand dollars ($40,000), while the appropriation in this State amounted to four thousand dollars ($4,000), but notwithstanding the township Board of Health had multiplied rapidly, and they hoped in the near future to establish a local Board in each township.

Prof. Harger thinks the bill, as had been presented, too complicated; better, one a milk bill, and the other a meat bill, but what is of first importance is stock inspection.

Dr. Schrieber thinks that any bill which would provide for the extermination of tuberculosis would be futile, unless it were so framed to necessitate the inspection of cattle from other States, thereby preventing the emigration of the disease.

Dr. Sallade thinks the general public should be better educated in tuberculosis and other contagious and infectious diseases, then there would be no trouble in getting laws passed to stamp out such diseases. That the Bovine Association, of which he is president, was formed for this purpose largely.

Dr. Zuill thinks that bills as presented are trying to the farmer and dairyman, as they had all to lose, even his time and annoyance.

Prof. Harger then offers the following resolutions:

*Whereas*, The sanitary inspection of the various food stuffs consumed by man has reached such dimensions as to become a question for public discussion, and knowing that their contamination with impurities is a menace to public health.

*Whereas*, We have undisputed evidence that certain diseases affecting dairy animals and their surroundings, and especially through the milk, as has been established in the case of tuberculosis of cattle and other diseases, and which are the source of widespread and fatal diseases among mankind. Be it

*Resolved*, That it is the sense of the Association that all dairies and their surroundings, the milk of which is offered for sale in this commonwealth, be thoroughly inspected, and that its members display every effort in securing proper legislation to procure a pure and wholesome milk supply.

Dr. Schauffler asks that this resolution be referred to a committee of three, but as it was presented as a general resolution it was on motion adopted as the sense of the Association, and that it be spread upon the minutes.

Report of committees now in order, Dr. Kooker, Chairman of Committee on Legislation, reported as follows:

To the President and members of the Pennsylvania State Veterinary Medical Association :

We beg to state that since the semi-annual meeting we have had no complaints of violation of the veterinary registry laws. The Keystone Association of this city appointed a committee to visit the Prothonotary's office and have the Veterinary Register revised according to law, viz.: have removals and deaths so recorded on the registry. We would advise the appointment by the President of this Association of a member in each county to examine the registry, and have it amended by noting the removals from the county and also those who may be deceased. We would like to have the sense of this meeting on the passage of a law requiring all those having diplomas and contemplating practicing veterinary medicine and surgery in this State be required to present the same before an examining board, which shall entitle them to be examined by said Board, and if the examination prove satisfactory then said board will issue a certificate to that effect, which will entitle them to be registered. We would like this subject discussed before this meeting, that the Legislative Committee may know the wishes of this Association in the matter of the bill as well as the passage of such a bill In the event of this Association agreeing with this committee then the Legislative Committee will have a bill drawn up according to their wishes, and ready for presentation at the semi-annual meeting in September.

We would also bring before the Association the matter of the meat and milk supply, and urge the profession to assist in every legitimate manner the Board of Health in its passage of such a bill. It is the opinion of this committee that the State Board of Agriculture that controls the contagious diseases of our live stock should be under the charge and control of veterinarians, and we would like the sense of the Association on the subject.

W. S. Kooker, Chairman,
T. B. Rayner,
Jas. W. Sallade,
Francis Bridge.

The report of the committee was received and ordered to be spread upon the minutes, when, on motion, a committee of three was appointed by the chair to draw suitable resolutions in regard to establishing of a State Board of Veterinary Examiners. This committee consisted of Drs. Zuill, Pearson and Kooker, who subsequently offered the following resolution, which was adopted:

*Whereas*, it is the sense of this Association that the registration bill fails to accomplish the purpose for which it was intended, that is, confining the practice of Veterinary Medicine to qualified practitioners, be it

*Resolved*, that the Legislative Committee of the Pennsylvania State Veterinary Medical Association of Pennsylvania be instructed to draft a bill, the object of which shall be the better regulation and practice of veterinary medicine, to provide a Board of Veterinary Examiners to examine all persons desiring to practice veterinary medicine in this State on the various subjects of their profession. The Board to be appointed as thereinafter provided, and to be known as the State Veterinary Examiner's bill.

W. L. Zuill,
W. S. Kooker.

It was further recommended that there be one member in each county which was represented in this Association appointed to look after the county registry of veterinarians.

It was moved and seconded that this Association send its Committee on Legislation to formulate a bill in connection with the meat and milk supply and report the same at the September meeting.

The following resolution was then offered by Dr. Williams, and, on motion, accepted and ordered to be spread upon the minutes:

*Whereas:* the veterinary profession has reached a stage in its development that may properly be designated independent, and is protected as an independent profession by the laws of the State of Pennsylvania, and

*Whereas:* We feel that it is within the power of the veterinarians of Pennsylvania to be of great public service to the owners and consumers of live stock and their products, be it

*Resolved:* That the control, eradication and investigation of contagious and other diseases among animals in this Commonwealth should be directed and carried out by veterinarians, who, indeed, are the only ones capable of serving the best interests of the public in the above-named capacity.

Chas. F. Williams.

The next report was that of the Committee on Sanitary Science and Police, by Dr. Formad. This notes no new diseases, speaks at some length of the disease of tuberculosis which was still existing, and of the value of tuberculin as a diagnostic agent in this disease, and of mallein in glanders, etc.

State Veterinarian Dr. Bridge, also a member of said committee, was asked to address the members upon the subject, which he did. He said that in the last year thirty-nine herds of cattle were reported in the State which suffered from tuberculosis, numbering altogether 450 head, 91 of which were diseased. Fifty-eight head were destroyed, which fully exemplified the disease, and in five head no visible traces of the disease appeared. The entire number had been tested with tuberculin, and of the 91 which showed some reaction all were destroyed. That 150 cases of glanders were reported; of this number 61 were found diseased, but that altogether 68 head had been destroyed. These also were tested with mallein. He further reported the outbreak, in two herds, of anthrax, of which six animals died.

The committee's report was accepted, and a vote of thanks extended the committee for the same.

There was no report from the Committee on Intelligence and Education, owing to the fact that a letter from the chairman, Dr. Weber, stated that on account of illness he was unable to be present and was unable to draft a report. This matter had been referred by him to Dr. Helmer, who also was unable to be present at the meeting.

The report of Corresponding Secretary Ridge was then read and on motion received and ordered to be spread upon the minutes.

### Report of Corresponding Secretary.

In the year 1887 this Association had twenty-nine members, and in that year took in two new members, but later one of these was expelled. In 1888 the Association took in three new members, one of these afterward being expelled. In 1889 we took in fourteen new members, of which three have been expelled.

In 1890 seventeen new members were admitted, of which one has been expelled.

1891 we added six new members, of which one has been expelled.

In 1892, like 1890, we added seventeen new members, while in 1893 we added to our membership twenty-eight new names, making our roll stand to-day 109 members in good standing. In 1892 we had sixty-four members, but the rapid increase of forty-five new members in these last two years shows that our Association is in a healthy condition, but have we anything to boast of, this being a State Association. We have only twenty-eight counties represented, while there are thirty-nine counties in this State that does not give us a member.

In Adam's County we have no members, while in Allegheny County we have five members. Armstrong, Beaver and Bedford give us none. In Berks we have three, Blair and Bradford none, while Bucks County gives us six. Butler, Cambria, Cameron, Carbon and Centre are not represented, but Chester thinks the Association a good institution and gives us seven good members.

Then we come to Clarion, Clearfield, Columbia and Cumberland, with no members, but Clinton gives us one and Crawford two.

Delaware four, Erie one, Elk none, Fayette one, Forest and Fulton none, while Franklin gives us four. Greene and Huntington has no members, but Indiana and Jefferson gives us one each. There is no member from Juniata, while Lackawanna and Lancaster has two members each. Lawrence none. Lebanon has two members and Lehigh gives us five. Luzerne gives us three, Lycoming one, while we have McKean, Mercer, Mufflin and Monroe without being represented. Montgomery gives us nine, Montour none, Northampton two, Northumberland one and Perry none, while old Philadelphia heads the list with thirty-seven. Pike and Potter has none, Schuylkill has two, Snyder, Somerset and Sullivan none, Susquehanna one, Tioga, Union, Venango and Warren none. Washington and Wyoming one each, but Wayne and Westmoreland has none, while the last county, York, gives us one.

The reprints of our last annual meeting were not received until after our semi-annual meeting, but were sent out as well as the semi-annual reprints. As our proceedings are published in our veterinary journals, we think this matter of sending out reprints useless expenditure.

Next in order the Treasurer's report was read.

PHILADELPHIA, MARCH 6th, 1894.

To President and members of the Pennsylvania State Veterinary Medica Association, I respectfully submit the following annual report as to the condition of the treasury for year ending March 6th, 1894:

| | |
|---|---|
| Cash in Treasury, March 7th, 1893 | $57 10 |
| Received as Membership fees | 131 00 |
| Received as dues | 74 00 |
| Received as extra assessment | 44 00 |
| Due by members as dues | 74 00 |
| Due by members as extra assessment | 44 00 |
| Due by members not qualified | 131 00 |
| Assets | $555 00 |

For year ending March 6th, 1894, on order drawn by the President and Secretary, the following bills have been paid to the respective orders:

| | |
|---|---|
| March 8th, 1893—To Dr. W. H Ridge, for postage | $38 00 |
| " " —To Dr. W. Horace Hoskins, for postage | 5 97 |
| " " —Rent of Hall and Janitor | 22 00 |
| March 13th, 1893—Loan from members | 125 00 |
| July 6th, 1893—Magee Bro., Printing | 13 50 |
| " " —C. H. Kimmig, filling certificates | 5 00 |
| July 13th, 1893—Magee Bro., printing By-Laws | 24 00 |

| | | |
|---|---|---|
| Aug. 14th, 1893—Magee Bro., printing application blanks | | 4 50 |
| Sept. 16th, 1893—W. Horace Hoskins, envelopes | | 2 75 |
| " " —Magee Bro., Printing programs | | 4 50 |
| " " —W. H. Ridge, expenses and postage | | 20 35 |
| March 6th, 1893—Due by members | | 249 00 |
| Total liabilities assumed and paid | | $512 57 |
| Balance in Treasury, March 6th, 1894 | | $42 53 |

JOHN R HART, Treasurer.

Approved by Auditing Committee:

W. G. Benher,
W. H. Knight,
L. O. Lusson.

There was no unfinished business.

No new business.

At this juncture Secretary Ridge read a letter of regret from Dr. Stanton.

Meeting adjourned to meet the following day, March 7th, 10 A. M. sharp.

---

PHILADELPHIA, PA., March 7th, 1894.

The second day's session of the Pennsylvania State Veterinary Medical Association was called to order by President Hoskins at 10.30 A. M. The following members were present: Drs. Allen, Benner, Collins, Ferley, J. C. Foelker, Formad, Gladfelter, Harger, John R Hart, Walter L. Hart, Hoskins, Houldsworth, Keely, Keil, Knight, Kooker, Lusson, W. B. E. Miller, McCarthy, Pearson, Phillips, Thos B. Rayner, Ridge, Sallade, Schaufler, Sturge, Tagg, Timberman, Webster, Williams, Zuill, Marsack, Adams, Felton, McAnulty, Nicholson, Rhoads.

As delegates from New Jersey Veterinary Medical Association, Drs. Dustan and Lockwood. As visitors from New Jersey Drs. Runge and Bailey; from Delaware, Dr. Eves; from Maryland, Dr Martenet. Also S. G. Hendren, E. L. Killner, A. Lallinger, R. M. Black, Dilher, S. K. Green, F. T. Shannon, F. H. Andrews, E. Mount, E. E. Terry, E. Knight, C. J. Marshall J. M Carter, R. J. S. Wicksel, C. E. Fouse, J. J. Rechtenwald, E. Hogg, B. M. Underhill, C. W. Bopd, M. L. Brackbill, George E. Harder, R. D. Heaton and C. F. Hartler.

A letter was read from Dr. Jas A. Waugh in which he regrets his absence and in which he calls attention of the Association to an advertisement in the "Spirit of the Times," (sporting paper) date, Dec. 3rd, 1893, of the Ontario Veterinary College, advertising for students for a term of three months. This letter was referred to the Board of Trustees.

The Chair says whereas the Wisconsin State Veterinary Medical Association of Graduates were this day in session, also considering kindred subjects to ours, that this Association should extend their congratulations, whereupon a motion to that effect prevailed, and Drs Ridge, Kooker and Timberman were appointed to compile a message of congratulations and forward the same to Wisconsin Association, which was answered by the Wisconsin Association in warm terms.

Delegates appointed to the various State Associations were then asked to report.

Dr. J. C. Foelker reported as delegate to the International Congress held at Chicago. Dr. Kooker, delegate to the New Jersey Veterinary Medical Association, reports that he was unable to attend. A similar report came from Dr. Timberman, who was a delegate to the New York State Veterinary Medical Association. Dr.

Lusson reported that he could not get the time and place of meeting to which he was appointed.

An application for membership of Dr. C. S. McKenna was read by the Secretary, and the same was referred to the Board of Trustees.

An Auditing Committee was appointed by the Chair to audit bills, vouchers, etc. Drs Benner, Lusson and Knight.

At this time a short recess was taken that the Board of Trustees and Auditing Committee might complete their work.

The Board of Trustees being prepared to report, Dr. C. S. McKenna was favorably recommended, whereupon he was elected to membership.

The Board also presented the following in reference to Dr. Waugh's letter:

To the Pennsylvania State Veterinary Medical Association: We beg leave to report that we have presented to us for consideration a letter from Dr. Jas. A. Waugh, calling our attention to an advertisement in the "Spirit of the Times," a sporting paper, dated Dec. 3rd. 1893, by the Ontario Veterinary College, advertising for students for a term of three months and that we deprecate the action of the Ontario Veterinary College, and recommend that this Association censure the Faculty of the said college for abbreviating the course of veterinary education.

The recommendations of the Board were received.

Finance Committee reports the Treasurer's report and bills correct.

Dr. Zuill's paper being called for, was read, entitled "Surgical Treatment of Lesions of the Hock," after which the Chair stated that if there were no objections he would ask for the reading of the paper by Dr. E Sturge on "Penetrant Cauterization in the Treatment of Lameness for Ostitis," as both papers covered much of the same ground, and the discussion would then take place on both papers. There being no objections, Dr. Sturge read his paper. The discussion following proved very interesting, critical and conflicting, and was participated in by Drs. Jno. W. Adams, W. B. E. Miller, Harger, Pearson, Eves of Delaware, Martenet of Maryland, Sallade, Hoskins, Foelker and Allen.

1.15 P.M adjourned for lunch.

The meeting reconvened at 2 30 P. M. The Board of Censons favorably recommended for membership Dr. C. S. McKenna, and on motion his election followed. The chair then called attention to the fact that the Comitia Minora of the United States Veterinary Medical Association would shortly meet to determine upon a place of meeting for "'94." He had been importuned by many that this State and Philadelphia be favored with this meeting, and it was now open for further consideration by the members. Many of the members strongly urged that the Association take active steps to win the meeting for Philadelphia, and it was afterwards moved and recommended and carried that the Chair appoint a Committee of three to draw up a suitable appeal and to ask their influence in winning the meeting for Philadelphia. The Chair answered that he would leave the appointment of this Committee to his successor in office.

Dr. Miller then followed with his paper on "Castration," which after reviewing the various methods, treated at length all the features connected with the performance of the operation with the animal standing He also explained the method adopted by Farmer Miles in operating upon Cryptorchids and answered fully many public criticisms made of his result in so operating. The paper was discussed by Drs. Pearson, Harger, Zuill and Breisacher and a motion offered by Dr. Pearson that a vote of thanks should be tendered Dr. Roger for stimulating Dr. Miller to prepare the paper just read, and was carried.

Dr. S. J. J. Harger then gave a very interesting paper on "Atmospheric Air in the Veins," recording a list of carefully performed experiments, with the results obtained.

This paper was discussed by Drs. Adams, Zuill and Breisacher.

The subject of "Transfusion of Blood," by Dr. Benner, was postponed until the September meeting.

Dr. E. Mayhew Michener was absent, and no letter having been received by the Secretary, there was no explanation of the absence of this paper.

Under reports of cases, Dr. Jas. T. McAnulty reported an interesting case of "Fracture of the Os-pedis."

The place for holding the semi-annual meeting was then considered, Harrisburg and Pittsburg being named. A vote being taken, Harrisburg was chosen.

The retiring President then introduced the President elect, Dr. Leonard Pearson. The latter on taking the Chair very warmly thanked the members for the high honor they had thus conferred.

The other officers elect were severally introduced, and each accepted the new duties allotted them.

After which a motion to adjourn prevailed.

ROBERT GLADFELTER, Recording Secretary.

---

Report of Chairman of Committee on Sanitary Science and Police:

Mr. President and Gentlemen :—As Chairman of your Committee on Sanitary Science and Police, I beg leave to offer you the following brief report:

Fortunately for the community we have not had any serious or extensive outbreaks of any contagious or infectious diseases, beyond the existence to a greater or lesser extent of those forms of contagious diseases which are ever present with us.

In importance bovine tuberculosis must necessarily be at the head of the list, from the influence which it exerts in the transmission of the disease to man.

We had several local outbreaks of glanders, only one of which, at Wilkesbarre, proved very disastrous in its results.

A local outbreak of Texas fever near our city has become a matter of record, but of the details and particulars I shall leave to my fellow-worker on the Committee, Dr. Francis Bridge, from whose hands they must come to you in a necessarily complete and official character, as he personally investigated that outbreak.

Tuberculosis is more prevalent near large cities than further away from them; more prevalent in dairy cattle than in other classes of cattle. Recent tests with tuberculin have shown that more than one-half of the dairy cattle of some herds are tuberculous, while in other herds the percentage of tuberculous cattle is very low and still other herds are entirely free from the disease.

Experienced veterinarians and breeders of cattle are of the opinion that tuberculosis in cattle is more prevalent now than it was a few years ago, which is seen in Europe as well, where it is corroborated by accurate statistics.

Many herds are so badly affected that the annual mortality destroys the entire profit from the remaining animals. In New York State whole herds have been examined by order of the State Board of Health and large numbers of animals have been slaughtered.

The agitation of the subject has so frightened the public that the consumption of milk has been greatly reduced in some places. It has also seriously affected the value of some breeds of cattle. For the protection of the farmer's interest, aside from the question of public health, it is important that some definite legislative action should be immediately taken, placing the control of tuberculosis in cattle, and the disposal of tuberculosis animals on a fixed basis, so that there may be uniformity of action in this respect, and so that the owners of animals may know what to do, what to expect and how to govern themselves accordingly.

Since it is at present manifestly impossible to make a thorough examination of every bovine animal in the State, it would be well to start the examination with an inspection of the dairies supplying milk to the cities. The direction of the inspection of animals and the control of their diseases should be in the hands of competent veterinary authorities, who should also issue a bulletin of information to cattle breeders, informing them of the dangers to be feared from this disease, and the hygienic and other measures to be adopted to restrict its progress and eradicate it from their herds. The veterinary inspector should have the power, under proper restrictions, to order the destruction of tubercular animals.

The question as to whether the owner should receive the whole value or part of the value or nothing is one which has received much attention.

To pay the full value for all tubercular animals would mean, as Dr. Lyman of the Massachusetts State Cattle Commission has pointed out for the State, to go into business of buying up tuberculous animals from all parts of this great country. for they would immediately be shipped to Pennsylvania almost as rapidly as fat cattle are now shipped to Chicago. On the other hand, if nothing were paid for these tuberculous animals, the owners would suffer an excessive, sometimes a ruinous loss in the interest of the public welfare. We might say that a tuberculous animal is worthless and thererore has no value, but we all know that they are sometimes very productive in the dairy, and frequently make beef of reasonably good appearance. The proper course and the just one would seem to be to arrange for the division of the loss between the owner and the commonwealth. A reasonable maximum value for the cattle for the various classes should be decided upon. The condemned cattle appraised within this limit by competent and unprejudiced appraisers. After their destruction the owner should receive a portion, say one-third or one-half of the appraised value.

Before closing my remarks on the subject of tuberculosis, I desire to revert to the value of tuberculin as a diagnostic agent, and that we are no longer justified in depending only upon methods of physical diagnosis in determining the existence of this disease in herds where we have found cases existing. Surely it would seem that we have just entered upon the real field of learning in regard to one of the probable means of the spread and propagation of tuberculosis. In a well-timed article recently read by Prof. Las. Law before the New York Veterinary Association and published in the last month's magazines, this question has been carefully and thoroughly discussed. We have been rudely awakened to a probable source of danger in this insidious disease. It seems that we have not been sufficiently impressed with the importance and influence of this disease. It may be the final explanation why the disease is so wide spread and so powerful in its results.

We probably have been looking too long for the direct transmission of the tubercle bacilli from parent to the offspring, and straining our eyes looking in the microscope for the existence of bacilli in the udder or in the milk, and have wholly lost sight of the general distribution of the poisonous products, which are also present in the animal economy. In other words we have been trying to discover in the body, in the secretions of the mammary gland, floating tubercle bacilli, or in the gland some localized tubercular masses, and apparently utterly ignorant of the fact that these same channels, which might bring the original bacilli to this point, were sending, distributing and revolving and attempting to eliminate through every excretory channel, the poisonous products that followed the introduction of these germs into the system.

In this connection, also, I would impress upon the members of the Association the value of mallein as an agent in the diagnosis of glanders, especially because there is no one disease which comes to us so clouded in obscurity as glanders, and which by physical signs more than severely tests the ability of an expert to decide. The use of mallein seems, as far as known, a perfectly safe test, but a much surer one than the common practice of testing by the inoculation of some other animal with the discharge of a questionable ulcer, or perhaps of a single farcy-like bud.

---

COMMITTEES OF PENNSYLVANIA STATE VETERINARY MEDICAL ASSOCIATION.

*Committee on Legislation.*—W. H. Hoskins, Chairman ; S. J. J. Harger, W. S. Kooker, J. C. McNeil, W. H. Ridge, J. W. Sallade, Chas. Schauffler.

*Committee on Sanitary Science and Police.*—M. E. Conrad, Chairman; Francis Bridge, C. R. Good, Jos. D. Houldsworth, C. C. McLean, B. F. Senseman. R. G. Webster.

*Committee on Intelligence and Education.*—J. W. Adams, Chairman; W. B. E. Miller, E. Stuge, Wm. Tagg, S. E. Weber.

---

### PENNSYLVANIA STATE VETERINARY MEDICAL ASSOCIATION.

The semi-annual meeting of the Pennsylvania State Veterinary Medical Association was convened at Chestnut Street Hall, Harrisburg, September 4th, at 10 A.M., with President Leonard Pearson in the Chair.

On roll call the following members responded to their names: Drs. Allen, Bachman, Benner, Bradley, Conard, J. C. Foelker, Good, Harger, John R. Hart, Hoskins, Keely, Keil, Kooker, Lintz, McNeil, Oyler, Pearson, Thomas B. Rayner, James B. Rayner, Ridge, Schauffler, Seltzer, Stanton.

As visitors: Dr. H. P. Eves, of Delaware, Secretary Edge of the Board of Agriculture, and Dr. E. Noeuck, of Reading, Pa.

After reading of the minutes, the President addressed the members in an excellent review of the past work and the present and future opportunities and need of the profession, specially attracting the members' attention to the need of better legislation looking to the live stock interests of our State.

The Board of Trustees reported favorably upon the applications of Drs. E. Noeuck, Thomas Castor, T. J. Kean, A. O. Koenig, C. J. Marshall, A. Sallinger, H. W. Turner, and Willard, and these were, afterward, unanimously elected to membership.

The application of Dr. Wm. T. S. Werntz was laid over until the March meeting of 1895.

The Secretary's report exhibited a very healthy condition of Association affairs, and that our growth was both vigorous and promising.

The reports of the various Committees were called for. The Committee on Legislation reporting the results of their deliberations, and offering for the consideration of the members the following two outlines upon the subjects of the need of the creation of a State Board of Live Stock Commissioners, and a Veterinary Medical Examiners' Board. These two bills were discussed by all the members present, and were referred back to the Legislative Committee, with instructions that they present them at the next session of the State Legislature. The Association voted them a sum of money to be used in completing the work necessary to be done in the submission of these bills for consideration at Harrisburg.

The report of the Committee on Sanitary Science and Police was offered by the Chairman, Dr. M. E. Conard, and was a review of the diseases prevailing in the State during the preceding six months, quoting in his report several outbreaks of "Anthrax," and one of foot-rot in sheep.

A telegram was received from Chairman Adams of the Committee on Intelligence and Education, informing the Association that he was detained at Trenton, and was unable to reach the meeting at all.

At the afternoon session the Association was favored with an excellent paper on "The Use of Mallein," by Dr. S. J. J. Harger. It was an exhaustive review of the value of this alkaloid as a diagnostic agent, and a resumé of the results obtained by all the leading experimenters who had given the subject the most consideration.

A very practical paper on "Eversion of the Uterus," referring specially to the bovine species, was presented by Dr. W. H. Ridge. It proved both interesting and instructive to all present.

Drs. Houldsworth and Irons being unavoidably absent, had very thoughtfully transmitted their papers on "Injuries to the Tongue of the Horse" and "Anthrax," and these were read by the Secretary.

During the afternoon Secretary Edge was accorded the privilege of the floor, and he addressed the Association upon Tuberculosis, and the laws needed for its better management and control, as well as the compensation to be accorded to the owners of stock where destroyed.

At 5.30 P. M. the meeting adjourned to meet at Philadelphia in March, 1895.

